# Mechanisms and otoprotective strategies of programmed cell death on aminoglycoside-induced ototoxicity

**DOI:** 10.3389/fcell.2023.1305433

**Published:** 2024-01-08

**Authors:** Lei Han, Zijing Wang, Daqi Wang, Ziwen Gao, Shaowei Hu, Dazhi Shi, Yilai Shu

**Affiliations:** ^1^ Department of Otorhinolaryngology, The Second Affiliated Hospital, Hengyang Medical School, University of South China, Hengyang, China; ^2^ ENT Institute and Department of Otorhinolaryngology, Eye and ENT Hospital, State Key Laboratory of Medical Neurobiology and MOE Frontiers Center for Brain Science, Fudan University, Shanghai, China; ^3^ Institutes of Biomedical Sciences, Fudan University, Shanghai, China; ^4^ NHC Key Laboratory of Hearing Medicine, Fudan University, Shanghai, China

**Keywords:** hair cell, ototoxicity, aminoglycosides, programmed cell death, gene therapy

## Abstract

Aminoglycosides are commonly used for the treatment of life-threatening bacterial infections, however, aminoglycosides may cause irreversible hearing loss with a long-term clinical therapy. The mechanism and prevention of the ototoxicity of aminoglycosides are still limited although amounts of studies explored widely. Specifically, advancements in programmed cell death (PCD) provide more new perspectives. This review summarizes the general signal pathways in programmed cell death, including apoptosis, autophagy, and ferroptosis, as well as the mechanisms of aminoglycoside-induced ototoxicity. Additionally, novel interventions, especially gene therapy strategies, are also investigated for the prevention or treatment of aminoglycoside-induced hearing loss with prospective clinical applications.

## 1 Introduction

Hearing loss (HL) is the most common sensory impairment in human beings. Almost 466 million people worldwide currently suffer from HL, and this figure is estimated over 900 million by 2050 (Olusanya et al., 2019). Aging, noise, infections, genetic defects, and long-term use of ototoxic drugs are the main causes of HL. Up to now, more than 150 ototoxic drugs have been documented, resulting in functional impairment and/or cellular degeneration in the inner ear (Tanaka et al., 2019). Aminoglycosides are one of the most common anti-inflammatory therapies used for Gram-negative bacteria in clinical practice, they are often selected as the first-line agents to treat suspected or confirmed severe acute infections (Pogue et al., 2020), cystic fibrosis (Smyth and Bhatt, 2014), and multidrug-resistant tuberculosis (Dillard et al., 2021) owing to their low cost and high efficacy. Despite various types of aminoglycosides are widely used in the clinic, including streptomycin, gentamicin, amikacin, neomycin, and kanamycin, there is a high risk of ototoxicity contributing to the degeneration of auditory cells in the inner ear. A growing body of evidence indicates that the loss of sensory hair cell (HC)s is the main cochlear pathology underlying drug-induced hearing loss, and hair cells are special non-regenerative cells in the inner ear (Shu et al., 2019), converting sound-induced vibrations into electrochemical signals. Damage or loss of hair cells leads to permanent hearing impairment (Fettiplace and Hackney, 2006). Aminoglycoside-induced hearing loss is typically cumulatively dose-dependent. Clinical studies have shown that 20%–47% of patients suffered from hearing loss due to the side effects of aminoglycoside-induced ototoxicity. In general, aminoglycosides primarily induce high-frequency hearing loss above 8,000 Hz, and gradually affect hearing loss at low-frequency with the time past (Wu et al., 2001; Crundwell et al., 2016; Sacks et al., 2018). Although numerous experiments have been done to mitigate the ototoxicity of aminoglycosides, no drug has been licensed for patients to prevent ototoxicity.

Programmed cell death (PCD), an active and orderly cell death mode, is a universal phenomenon in the development of organisms (Nagata and Tanaka, 2017). Abnormal regulation of PCD is closely related to a series of human diseases, such as immune diseases (Günther et al., 2013), neuropsychiatric disorders (Margolis et al., 1994), and cancer (Carneiro and El-Deiry, 2020). Previous studies have reported that ototoxic drugs can cause programmed death of auditory hair cells, resulting in hair cell loss and hearing damage (Wu et al., 2020). For a better understanding of the mechanism of aminoglycoside-induced ototoxicity, it is necessary to investigate the different forms of hair cell programmed death, which may eventually provide new ideas for the prevention and treatment of drug-induced hearing loss. In this review, we collected the latest insights into protecting hair cells from the ototoxic effect of aminoglycosides by regulating PCD pathways.

## 2 The mechanisms of aminoglycoside ototoxicity

Over the past few decades, great progress has been made in elucidating the mechanisms of ototoxicity induced by aminoglycosides. Biological analysis has shown that the formation of oxidative free radicals and subsequent triggering of PCD plays a crucial role in hair cell death. Understanding the way aminoglycosides enter into hair cells and the role of free radicals, as well as how PCD contributes to irreversible HL is helpful to develop new therapeutic methods to protect hearing.

### 2.1 Trafficking of aminoglycosides into the sensory hair cells

To date, aminoglycosides are clinically available therapies for systematic or local administration to the inner ear. The endothelial cells of cochlear blood vessels are coupled together by tight junctions to form the primary blood–labyrinth barrier (BLB), separating the cochlear cells and fluids from the bloodstream (Nyberg et al., 2019). Aminoglycoside transmission in the inner ear, it is much easier to traverse into the BLB of the stria vascularis in comparison to the spiral ligament of the adjacent perilymphatic position (Dai and Steyger, 2008). Up to now, the mechanisms of the aminoglycosides passing through the BLB, i.e., endothelial cells, the vascular texture, and the marginal cells to the endolymph are still unclear (Koo et al., 2015). Kim et al. showed that megalin was a candidate carrier of the aminoglycosides in the stria vascularis in a study of an *in vivo* real-time tracking procedure, showing how the aminoglycosides transported across the blood–labyrinth barrier, and blockade of megalin by inhibitor cilastatin could prevent drug accumulation in the inner ear (Kim and Ricci, 2022) (Table 1). There are several pathways for aminoglycosides to permeate HCs from the endolymph. Ion channels transport, especially mechanoelectrical transducer (MET), plays a vital role in the uptake of aminoglycosides to HCs (Vu et al., 2013; Nilius and Szallasi, 2014). The MET channel is a nonspecific cation channel at the tip of hair cells stereociliary with high calcium permeability. Its narrowest portion is at least 1.25–1.5 nm in diameter, sufficient to allow aminoglycosides to enter hair cell cytoplasm (Farris et al., 2004; Alharazneh et al., 2011). Blocking MET channels (e.g., with ORC-13661, d-Tubocurarine, or UoS-7692) prevents aminoglycosides entry into hair cells and confers hair cell protection from aminoglycosides (Kirkwood et al., 2017; Kitcher et al., 2019; Kenyon et al., 2021). It is worth mentioning that long-term blocking of the MET channel would affect hair cell function and worsen hearing impairment. In addition to MET channels, other ion channels are also associated with the uptake of aminoglycosides into the HCs; for example, transient receptor potential (TRP) channels, which are highly expressed in hair cells and play a key role in aminoglycosides entering into hair cells (Myrdal and Steyger, 2005; Lee et al., 2013). The TRPA1 channel is a member of the TRP family, which is a non-selective cation channel activated by certain pungent compounds and lipid peroxidation (Balestrini et al., 2021). In the mammalian cochlea, blocked the MET channel, and activated TRPA1 channels promote the uptake of aminoglycoside. Therefore, it has been proposed that excessive oxidative stress activates TRPA1 channels to enhance the uptake of aminoglycosides by damaged hair cells (Stepanyan et al., 2011).

**TABLE 1:** Potential drug targets for the treatment of Aminoglycoside ototoxicity.

Regulatory pathway	Mechanism	Compound	References
Targeting Alternative Entry Routes	Inhibit aminoglycosides entry into the endolymph	Cilastatin	Kim and Ricci (2022)
	MET channel blocker	ORC-13661	Kitcher et al. (2019)
		d-Tubocurarine	Kirkwood et al. (2017)
		UoS-7692	Kenyon et al. (2021)
Targeting Oxidative Stress Pathway	Antioxidant	d-Methionine	Fox et al. (2016)
		Vitamins C	Gong et al. (2022)
		N-acetylcysteine (NAC)	Aladag et al. (2016)
		Quercetin	Hirose et al. (2016)
		Galangin	Kim et al. (2016)
		Fursultiamine	Kim et al. (2021)
		Salicylate	Sha et al. (2006)
Targeting Apoptosis Pathway	Caspase inhibitor	z-VAD-FMK	Matsui et al. (2003)
		z-LEHD-FMK	Okuda et al. (2005)
	JNK inhibitor	D-JNKI-1	Eshraghi et al. (2007)
		Estradio	Nakamagoe et al. (2010)
	Nrf2 agonist	Ebselen	Kil et al. (2017)
		Apigenin	Jia et al. (2022)
		Heme oxygenase-1	Yang et al. (2022)
	YAP agonist	XMU-MP-1 (XMU)	Wang et al. (2022)
Targeting Autophagy Pathway	MTOR inhibitor	Temsirolimus (CCI-779)	Ye et al. (2019)
Targeting Ferroptosis Pathway	Iron chelator	2,3-Dihydroxybenzoate	Song and Schacht (1996)
		Deferoxamine	Song and Schacht (1996)
	Selective ferroptosis inhibitor	Liproxstatin-1	Zheng et al. (2020)

**FIGURE 1:**
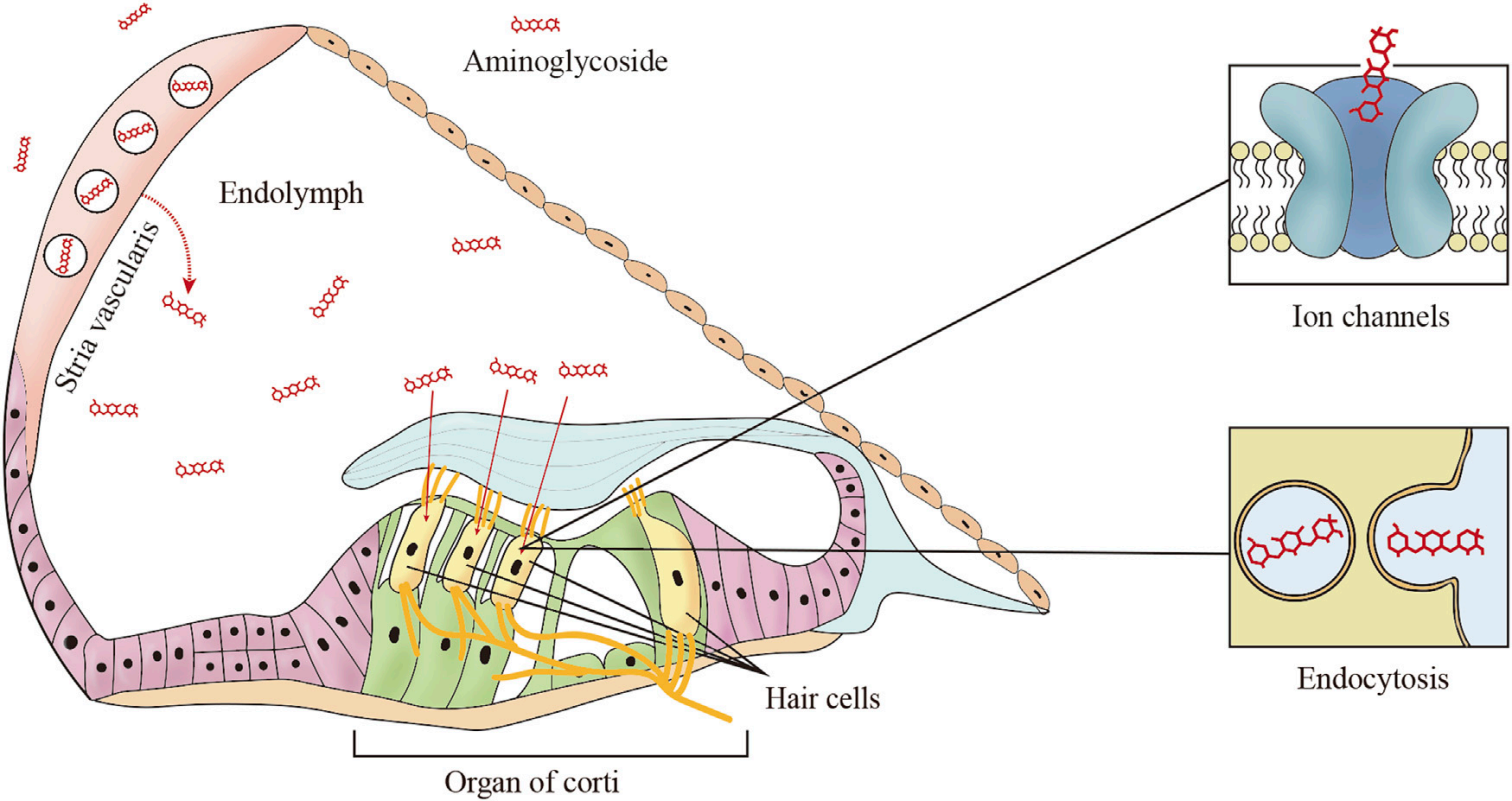
The main trafficking routes of aminoglycosides. In systemic administration, aminoglycosides are trafficked from cochlear capillaries into the endolymph, and then aminoglycosides enter the hair cells from the endolymph by two common mechanisms including ion channels and endocytosis.

Endocytosis is another route by which aminoglycosides enter the apical membrane of hair cells, although there is no direct evidence that endocytosis is involved in aminoglycoside-induced cytotoxicity (Hashino and Shero, 1995). It is worth mentioning that Breglio et al. revealed that HSP70-dependent paracrine protection of hair cells is mediated by exosomes which are generated by the direct outward budding of the plasma membrane during endocytosis (Breglio et al., 2020). Targeting endocytosis is regarded as a strategy to prevent ototoxicity is still challenging, because it is not the primary mechanism of aminoglycoside-induced ototoxicity (Figure 1).

### 2.2 Free radical accumulation and programmed cell death

Aminoglycosides rapidly accumulate in the cytoplasm, mitochondria, and endoplasmic reticulum (ER) after entering the cell via the above channels. Aminoglycosides inhibit protein synthesis and increase mRNA misreading by interacting with ribosomes, leading to the accumulation of misfolded proteins and causing various cellular stresses. Excessive reactive oxygen species (ROS), produced by cellular metabolism or contact with foreign substances, have been detected in cochlear tissue immediately after cellular stresses (Navarro-Yepes et al., 2014). ROS is an important group of free radicals, which can cause direct damage or act as key mediator signaling molecules, resulting in a series of biological effects, including caspase3 activation, which promotes hair cell programmed death (O'Sullivan et al., 2017). Various forms of PCD lead to different degrees of biological changes in hair cells. When autophagy occurs, bilayer organelles called autophagosomes bring cytoplasmic products to the lysosomes for degradation. In contrast to autophagy, cells undergoing ferroptosis exhibit increased lipid peroxidation, shrunken mitochondria, and increased mitochondria membrane density (O'Sullivan et al., 2017). It is generally accepted that the most common form of programmed death induced by aminoglycosides in hair cells is apoptosis, which is characterized by cell shrinkage and chromatin agglutination. In preclinical models, antioxidants were used to reduce aminoglycoside-induced ROS production, suppress programmed cell death, and increase cell viability. Antioxidants applied in the study of ototoxicity of aminoglycosides, including D-methionine (Fox et al., 2016), Vitamin C (Gong et al., 2022), N-acetylcysteine (Aladag et al., 2016), quercetin (Hirose et al., 2016), galanin (Kim et al., 2016), fursultiamine (Kim et al., 2021), and salicylates (Sha et al., 2006).

## 3 Apoptosis pathways in aminoglycoside-induced ototoxicity

**FIGURE 2:**
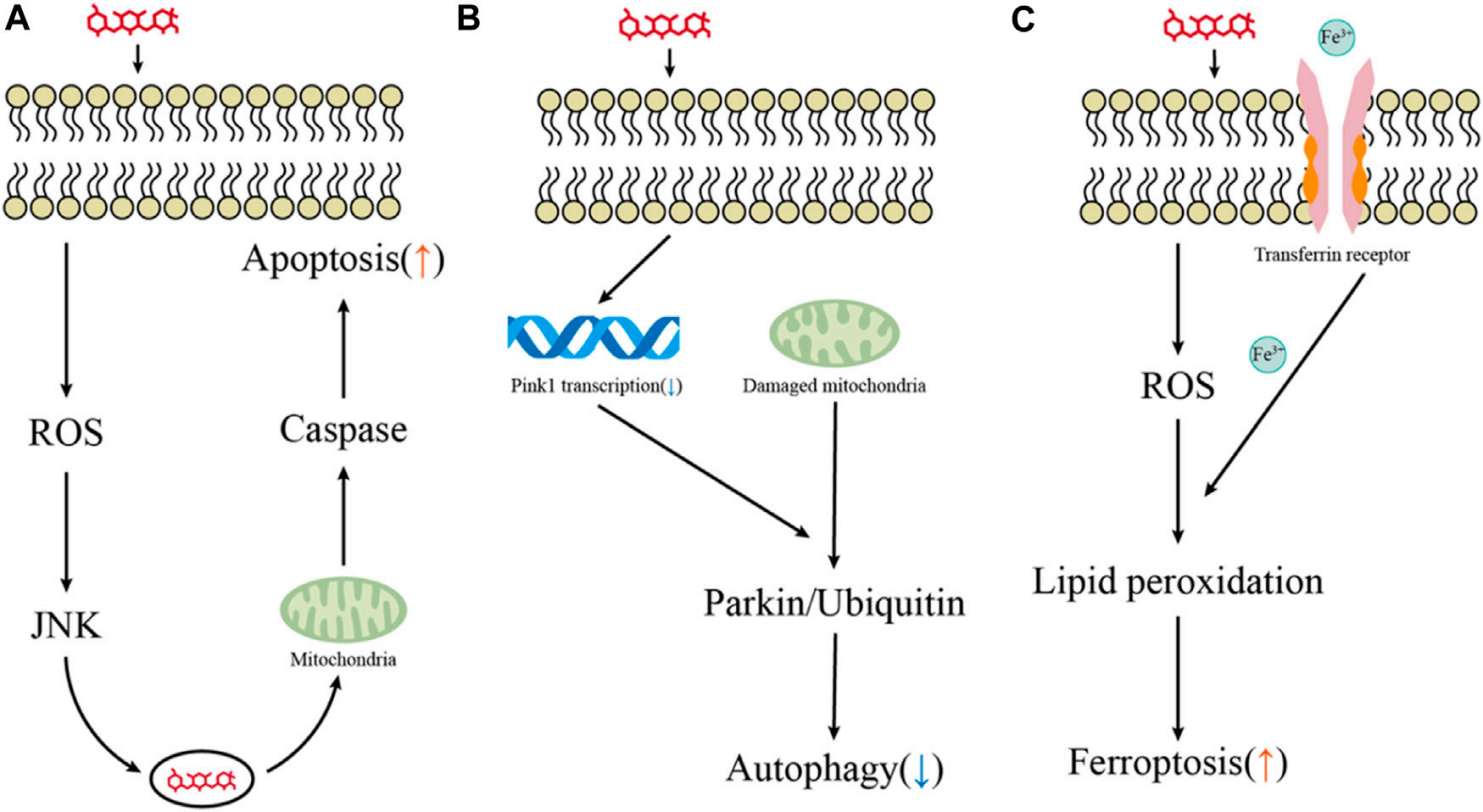
Key pathways for hair cell death induced by aminoglycosides. **(A–C)** Major pathways of aminoglycoside-induced hair cell apoptosis, autophagy, and ferroptosis.

Apoptosis is a tightly controlled process by multiple genes. These genes are very conserved among species, such as the Bcl-2 family, caspase family, and tumor suppressor gene P53 (Igney and Krammer, 2002; Szegezdi et al., 2006). External and internal stimuli, in combination with extrinsic and intrinsic apoptosis apoptosis-related genes, can induce cell apoptosis. Bodmer et al. revealed that gentamicin does not cause apoptosis through the Fas receptor, which is the best-characterized member of the extrinsic pathway family and a receptor signal that must be activated during the extrinsic process of apoptosis. This suggests that hair cell apoptosis triggered by aminoglycosides may be initiated primarily through an intrinsic pathway in response to stress signals and excessive ROS (Bodmer et al., 2003) (Figure 2).

### 3.1 Apoptosis mediated by caspase

Caspase activation is well known as a common intrinsic pathway for drug-induced hair cell apoptosis. Functionally, caspases involved in apoptosis have been subclassified into initiator (caspases 8, 9, and 10) and effector (caspases 3, 6, and 7) caspases, the initiator caspases are activated by proapoptotic signals and subsequently coordinate their activities to activate effector caspases (Brenner and Mak, 2009; McIlwain et al., 2013). Caspase-9 is considered the major initiator caspase that participates in aminoglycoside-induced hair cell death, which activates downstream caspase-3 and demolishes key structural proteins, ultimately leading to apoptotic of hair cells (McIlwain et al., 2013). It has been reported that caspase-9 and caspase-3 expression was elevated in both HEI-OC1 cells and cochlear exposed to aminoglycosides, and the use of caspase-9 specific inhibitor (z-LEHD-FMK) or general caspase inhibitor (z-VAD-FMK) was found to effectively reduce the expression of caspase and prevent HC death (Matsui et al., 2003; Okuda et al., 2005). Notably, caspase 8 was found to cooperate with RIP kinase to regulate a type of regulatory cell death—necroptosis. It was found to be involved in the ototoxicity of aminoglycosides *in vivo* (Ruhl et al., 2019), but, the mechanism of programmed necrosis in aminoglycoside-induced ototoxicity is poorly understood.

### 3.2 The c-jun NH2-terminal kinases (JNKs)

As a redox-sensitive factor, the c-jun NH2-terminal kinases (JNKs), an essential member of the mitogen-activated protein kinase superfamily, play a vital role in the cellular antioxidant self-defense system (Shen and Liu, 2006). Upon aminoglycoside insult, the JNK pathway is activated by phosphorylation and increases the expression of downstream target genes, including itself, to promote hair cell apoptosis (Ylikoski et al., 2002). At the molecular level, cyclin-dependent kinases (CDKs) regulate aminoglycoside-induced hair cell apoptosis by interfering with the JNKs pathway, inhibition of CDK2 activity by pharmaceutical or CDK2 knockout affects JNK signaling and improves resistance to the ototoxicity of gentamicin (Tao and Segil, 2022). Furthermore, small molecule drugs such as a peptide inhibitor (i.e., D-JNKI-1) (Eshraghi et al., 2007) and estradiol (Nakamagoe et al., 2010) were discovered to attenuate hair cell loss following aminoglycoside administration by interfering JNK pathway.

### 3.3 The role of nuclear factor erythroid 2- related factor 2 (Nrf2) in apoptosis

Nuclear factor erythroid 2-related factor 2 is a transcription factor encoded by the NFE2L2 gene (Sykiotis and Bohmann, 2010), it is localized in hair cells and supporting cells in the human Corti’s organ (Hosokawa et al., 2018). Nrf2 is an important antioxidant regulator in a series of chronic toxic lesions caused by oxidative stress. In recent years, studies on the relationship between Nrf2 transcription factors and hearing impairment have given people a new understanding of hair cell damage and provided new strategic targets for the development and reuse of new drugs (Cuadrado et al., 2019).

Nrf2 is regulated by upstream pathways, Baird et al. revealed that Sestrin-2 (Sesn2), a member of the antioxidant family, is involved in protecting hair cells against gentamicin by activating Nrf2 (Baird and Dinkova-Kostova, 2011). Appreciation that Nrf2 pathway activation provides hair cells protection from ototoxic drugs, the protective effect on hair cells seems to be heading in a new trend, that is, by stimulating the expression of Nrf2 and preventing hair cells from being damaged. Ebselen is a potent glutathione peroxidase (GPx) mimic and inducer with antioxidant and anti-inflammatory effects. It has been found to attenuate aminoglycoside-induced ototoxicity by activating the Nrf2 signaling pathway, increasing glutathione, and dramatically stimulating GPx1 transcription (Gu et al., 2021). Encouragingly, the safety and efficacy of ebselen for the prevention of noise-induced hearing loss are undergoing phase 2 clinical trials (Kil et al., 2017). Moreover, other drugs have also been found to protect hair cells from aminoglycosides by intervening in the NRF2 pathway, such as apigenin (Jia et al., 2022) and Heme oxygenase-1 (Yang et al., 2022).

### 3.4 Other apoptosis signals

Other pathways have been investigated for cellular apoptosis. The Bcl-2 protein family could be the main regulator that triggers caspase activation. From a functional point of view, Bcl-2-related proteins can inhibit or accelerate cell apoptosis, while the interaction between the two opposite proteins is a key factor in determining cell death (Willis et al., 2003). Studies have shown that overexpression of the anti-apoptotic Bcl-2 gene may have a certain inhibitory effect on hair cell apoptosis induced by aminoglycosides (Liu et al., 2007). Another apoptosis signal, transcription factor forkhead box O3 transcription factor (Foxo3), can regulate the expression of stress response proteins in the body and participate in the apoptosis of various tissues (Dansen and Burgering, 2008; Gargini et al., 2015). The role of the Foxo3 gene and Bcl-2 family genes in apoptosis was further confirmed by the discovery that Wnt/β-catenin signaling regulates Foxo3 and Bcl-2 expression, controls the content of ROS, inhibits apoptosis and protects HCs immune from neomycin injury (Liu et al., 2016). Interestingly, the Hippo/Yes-associated protein (YAP) signaling pathway, which plays a key role in the development and progression of cancer (Harvey and Tapon, 2007), may be involved in hair cell damage induced by aminoglycosides. Wang et al. regulated the Hippo/YAP signaling pathway *in vitro* against neomycin-induced HC loss by inhibiting cell apoptosis and decreasing ROS accumulation by using the YAP agonist XMU-MP-1 (XMU) successfully (Wang et al., 2022). Inhibition or activation of numerous signaling pathways should be considered when regulating apoptosis to better protect hair cells from ototoxic damage.

## 4 Autophagy pathways in aminoglycoside-induced ototoxicity

Substantial findings suggest that cellular death mediated via autophagy is essential in hearing loss. Autophagy is considered an orderly degradation and recycling mechanism, which decomposes unnecessary or dysfunctional cellular components to maintain the homeostasis of the body (Eskelinen, 2019). In the early stage of the disease, autophagy activation may facilitate the disease process delay, nevertheless, cells may over-activate autophagy leading to cell death as the prominent pathogenic factors appear (Wu et al., 2020). Thus, in the growth and maintenance of multicellular organisms, autophagy always keeps a delicate balance, and loss or overactivation of autophagy regulation could result in the occurrence of sickness (Levine and Kroemer, 2008; Narendra et al., 2009). Previous experiments proved that autophagy induction increases the survival of retinal ganglion cells (RGCs) (Rodríguez-Muela et al., 2012). However, the effect of the activation of autophagy on the ototoxicity of aminoglycosides remains controversial.

### 4.1 Autophagy activation protects hair cells from aminoglycoside-induced ototoxicity

Combined injection of kanamycin and furosemide can cause capillary detachment and reduce the autophagic flux for lysosome production, leading to the degeneration of hair cells and spiral ganglion neurons. After autophagy dysfunction was partially ameliorated with an MTOR inhibitor temsirolimus (CCI-779), lysosome defects were significantly relieved, oxidative stress levels were reduced, and the density of surviving spiral ganglion neurons and hair cells was significantly increased (Ye et al., 2019). He et al. stimulated the autophagy activity with rapamycin, a commonly used autophagy activator, and significantly reduced the ROS levels, apoptosis, and cell death after neomycin or gentamicin injury, and the viability of cochlear explants and HEI-OC1 cells was enhanced. In contrast, exposure to aminoglycosides resulted in reduced autophagy activity, increased ROS levels, apoptosis, and cell death following treatment with the autophagy inhibitor 3-methyladenine (3-MA) or knockdown of autophagy-related (ATG) proteins (He et al., 2017).

PINK1 is a serine/threonine kinase located in the mitochondria, while Parkin is an E3 ubiquitin ligase present in the cytoplasm. PINK1 recruits parkin to depolarized mitochondria, ubiquitinates the mitochondrial substrates, and drives autophagy (Lazarou et al., 2012; Goiran et al., 2018; Pickles et al., 2018). Yang et al. treated HEI-OC1 cells and mouse cochlear hair cells with 400 µM gentamicin and found that gentamicin exposure promoted the PINK1 degradation and parkin recruitment, enhancing autophagy activity. They interfered with PINK1 expression of HEI-OC1 by using specific PINK-siRNA and demonstrated that activation of PINK1 prevented gentamicin-induced damage by promoting spontaneous autophagic and inhibiting the increase of p53 in HEI-OC1 cells (Yang et al., 2018). The transcription of PINK1 is mainly regulated by ATF3 (activating transcription factor 3), XBP1 (X-box binding protein 1), and FOXO3 (forkhead box O3) (Bueno et al., 2018). By inducing ATF3 expression in HEI-OC1 cells and cochlear hair cells, neomycin repressed Pink1 transcription and decreased autophagic activity (Zhang et al., 2023). Accumulating evidence suggests that gene modulation or pharmacological intervention of autophagy may have therapeutic potential for aminoglycoside-induced hearing loss (Figure 2).

### 4.2 New perspective on the role of autophagy in ototoxicity of aminoglycosides

Some scholars have proposed that appropriate ROS after neomycin injury can promote autophagy, restore damaged cellular components, and maintain the stability of the internal environment. However, the role of autophagy in aminoglycoside ototoxicity is controversial, in a large, unbiased screen, Ryan and others found that autophagy is involved in the ototoxic injury of hair cells in a complex manner, they predicted that many complexes protect hair cells from aminoglycoside damage could inhibit autophagy, in contrast, some complexes could promote autophagy to reducing ototoxicity (Draf et al., 2021). Recently, Bruijn et al. reported that aminoglycosides could trigger the interaction of RIPOR2 in murine hair cells with GABARAP, an autophagy pathway that plays an important role in clearing and restoring dysfunctional cellular components (de Bruijn et al., 2020). Surprisingly, the reduction of RIPOR2 and GABARAP proteins completely inhibited aminoglycoside administration-induced hair cell death and subsequent hearing loss. In addition, disrupting the autophagy pathway by canceling PINK1 or Parkin expression could also protect hair cells from aminoglycoside-induced ototoxicity (Li et al., 2022). It would be interesting to explore the role of autophagy in aminoglycoside-induced ototoxicity, as both high and low levels of autophagy can cause damage to the hair cells, and the degree of autophagy requires exquisite control. As a possibility, the effects of autophagy on hair cell function and survival are multi-pathway regulated, and the level of autophagy needs to be precisely adjusted to protect hair cells from aminoglycosides.

## 5 Ferroptosis pathways in aminoglycoside-induced ototoxicity

Ferroptosis is a new type of iron-dependent programmed cell death, which is different from apoptosis, necrosis, and autophagy (Dixon et al., 2012; Galluzzi et al., 2018; Shen et al., 2018). Three essential hallmarks define ferroptosis: oxidation of polyunsaturated fatty acid-containing phospholipids, loss of lipid peroxide repair, and reduction in the accumulation of redox-active iron (Friedmann Angeli et al., 2014; Xie et al., 2016; Wu et al., 2021).

Iron has a great influence on the metabolism of cells and plays an important role in tissue damage, as it participates in the generation of highly reactive oxygen species. Two iron chelators, deferoxamine, and 2,3-dihydroxybenzoate could compete with gentamicin for “free” iron ions and reduce ototoxic damage to hair cells (Song and Schacht, 1996). Considered that “free” iron ions are crucial members in the occurrence of ferroptosis. Therefore, established iron chelates may be selected as promising therapeutic agents, reducing aminoglycoside-induced ototoxicity by inhibiting ferroptosis.

Increasing attention has been attracted to further research investigating the pathophysiological role of ferroptosis in the field of drug-induced hearing loss (Figure 2). Ferroptosis may coordinate with other types of cell death involved in drug-induced hair cell ototoxic damage, and recent studies have provided that activation of the autophagy pathway promotes ferroptosis by degrading ferritin (Hou et al., 2016). Mei et al. found that HEI-OC1 cells treated with cisplatin not only markedly augmented iron accumulation but reduced the activity of glutathione peroxidase 4 (GPX4), an intracellular antioxidant enzyme that inhibits the production of lipid peroxidation (Mei et al., 2020). Notably, treatment with the specific ferroptosis inhibitor ferrostatin-1 could effectively reduce cisplatin-induced ototoxicity by inhibiting lipid peroxide free radicals and improving the mitochondrial function of hair cells (Hu et al., 2020; Mei et al., 2020). Like cisplatin, ferroptosis could be induced in HEI-OC1 cells and neonatal mouse cochlear explants following aminoglycoside exposure. Pretreatment with selective ferroptosis inhibitor liproxstatin-1 (Lip-1) significantly alleviated the production of ROS and the destruction of mitochondrial membrane potential (ΔΨm) in the HEI-OC1 cells (Zheng et al., 2020).

Ferroptosis is a newly discovered hair cell programmed death associated with aminoglycoside-induced ototoxicity, and inhibition of ferroptosis successfully prevented hearing loss induced by cisplatin and aminoglycosides *in vitro* model. However, the role of ferroptosis in aminoglycoside-induced ototoxicity has not been studied in mammals *in vivo*, and the mechanisms of ferroptosis in regulating ototoxic hair cell death are unclear. We need more studies to understand the impact of ferroptosis on ototoxicity. Additionally, we need to extend this research to animals to promote ferroptosis inhibitors as possible ear protection drugs for patients.

## 6 Gene therapy regulates programmed cell death induced by aminoglycosides

The past few decades witnessed the development of gene therapy, which focuses on providing effective gene interventions in different periods to treat or prevent diseases (Omichi et al., 2019; Zhang et al., 2020). Gene therapy candidates are probably the most complex drugs invented by mankind to date and may become one of the important means to treat intractable diseases (Maeder et al., 2019; Santiago-Fernández et al., 2019; Wu et al., 2019; Stadtmauer et al., 2020). Applying this technology to the clinic is still challenging, but the trends are encouraging in the future.

### 6.1 Overexpression of apoptotic genes

At present, it is possible to construct regulatory elements upstream of the target gene by artificial modification, so that the gene can be transcribed and translated under artificially controlled conditions to achieve the products of gene overexpression.

Several neurotrophic factors, especially brain-derived neurotrophic factor (BDNF) and neurotrophin-3 (NT-3) promote the growth, survival, and interconnectivity of hair cells (Ramekers et al., 2012). In animals exposed to kanamycin, infusion of NT-3 or BDNF into the perilymphatic space of the cochlea was found to prevent the loss of auditory hair cells (Ruan et al., 1999). Therefore, neurotrophin gene transfer into the inner ear may be a practical means to rescue ototoxin-exposed cochlear hair cells. Indeed, adenovirus-mediated neurotrophic factor overexpression in the inner ear demonstrated that hair cells and spiral ganglion neurons are protected from degeneration and death caused by aminoglycoside-induced ototoxicity (Kawamoto et al., 2003; Wise et al., 2010; Leake et al., 2020). Not only neurotrophins but also several apoptosis-related genes have been amplified by overexpression *in vivo* to observe if they can mitigate drug-induced ototoxicity. In the auditory system, overexpression of Bcl-2, an essential gene involved in hair cell apoptosis mentioned above, has been shown to protect against aminoglycoside-induce ototoxicity *in vitro* and *in vivo* mouse models (Pfannenstiel et al., 2009). Utilized adenovirus-mediated gene therapy overexpressing human catalase in the inner ear and demonstrated that overexpression of antioxidant genes could significantly protect the HC from ototoxic damage (Kawamoto et al., 2004). In preclinical studies, an increasing number of genes are amplified in mice by overexpression to prevent the occurrence and development of diseases. In the future, virus-mediated gene overexpression is expected to solve the disease problem in clinical practice.

### 6.2 Knockdown of apoptotic genes

CRISPR/Cas9 is a novel gene editing technology. Its simplicity, ease of use, and powerful gene editing capabilities have rapidly attracted substantial attention from scientists in different fields (Deltcheva et al., 2011; Cong et al., 2013). This system has been applied to the mouse model of human congenital deafness in preclinical studies multiple times and successfully corrected the gene mutation in the inner ear of mice to rescue their hearing (Gao et al., 2018; Xue et al., 2022). It is heartening to see the remarkable success in the prevention and treatment of acquired sensorineural hearing loss using CRISPR/Cas9 gene editing technology recently.

Inhibitor of apoptosis protein (IAP) prevents apoptosis by blocking canonical caspase-mediated apoptosis and JNK signaling pathways (Deveraux et al., 1999). In many cases, IAP family proteins can cross species barriers to inhibit apoptosis, this means that although the details of their regulation may differ, these proteins target a common mechanism of programmed cell death (Deveraux and Reed, 1999). X-linked inhibitor of apoptosis protein (XIAP) is one of the most effective IAP, it does not prevent caspase activation but inhibits them after they are activated, this post-activation inhibition occurs because XIAP binds to neo-epitopes that are exposed when caspases are activated by cleavage (Unsain et al., 2013). Sun et al. confirmed that XIAP was involved in HCs apoptosis. Injecting neomycin into high-expressing XIAP mice, they found that overexpression of XIAP effectively prevented the loss of HCs, especially in the apical turn (Sun et al., 2014). Whereas, XIAP in hair cells after exposure to aminoglycosides may be degraded by upstream pathways regulation. HtrA2 (High-temperature demand A2)/Omi is a nuclear-encoded protein found in the inner mitochondrial membrane space. In response to apoptotic stimuli, HtrA2 is released into the cytoplasm, cleaving XIAP, Apollon/BRUCE, and other proteins to promote apoptosis and maintain homeostasis (Goo et al., 2014). Our team tried to inhibit XIAP degradation to resist apoptosis by knocking down *Htra2* with the application of the CRISPR-Cas9 system. We targeted editing of the *Htra2* gene and demonstrated that knockout of the *Htra2* gene by the adeno-associated virus (AAV)-mediated CRISPR/Cas9 system could effectively prevent aminoglycoside-induced ototoxic deafness in mice. The protection against neomycin exposure *in vivo* after pretreatment with the AAV-CRISPR/Cas9 system can last for a long time, even up to 8 weeks after the injection system. We also evaluated the safety of AAV-CRISPR/Cas9 systems, during the observation period of the study, no obvious off-target conditions were found, and there was no effect on the hearing of wild-type mice, indicating that the treatment system was safe (Gu et al., 2021). The CRISPR/CasRx system, a member of the CRISPR system type VI, provides an efficient and specific new method for RNA manipulation in both prokaryotes and eukaryotes (Shmakov et al., 2015; Abudayyeh et al., 2016; Smargon et al., 2017). After using the AAV-CasRx-gRNA system, we found that knocking out the *Htra2* gene transcript could effectively reduce the loss of cochlear hair cells after neomycin exposure and attenuate hearing loss in mice. In addition, knockdown of the *Htra2* gene significantly reduced the mRNA expression of Casp3 and Casp9 in cochlear hair cells after neomycin treatment (Guo et al., 2022). These results demonstrated that hair cell ototoxic damage can be effectively prevented by the specific knockout of a target gene at the DNA/RNA level. Recent clinical studies have shown that RNA interference therapy is an available option to target and suppress genes associated with cancer, and accelerate the development of cancer treatments in a novel way (Kara et al., 2022). What’s more, studies have been detecting the RNA interference (RNAi) as a way of selectively suppressing mutant alleles in animal models with genetic hearing loss (Shibata et al., 2016). Therefore, it is of great interest to investigate the treatment of RNA interference as a promising strategy for preventing drug-induced hearing loss in the future.

In most studies of protective therapy, drugs are administered intraperitoneally, subcutaneously, or intravenously. After entering the body, many small-molecule therapeutic drugs cannot play a role due to short plasma half-life or unable to reach inner ear cells through the blood–labyrinth barrier. Compared with drug administration, local injection of gene therapy agents into the inner ear owns several outstanding advantages (i) the therapeutic effect of gene therapy could achieve a long-term disease prevention and treatment, (ii) personalized gene therapy could achieve the curative effect based on the individual genetic status and treatment condition, (iii) gene therapy has the potential to correct defective genes for the treatment of diseases fundamentally. Further evaluation is necessary for the safety and application of gene therapy, we believe that shortly, gene therapy can be used as an accurate treatment for hearing loss, restoring hearing function and preventing the development of hearing loss in a way that conventional medicine cannot.

## 7 Conclusion

Ototoxic drugs enter hair cells via the endolymph and accumulate in organelles to produce excessive ROS. The accumulation of ROS will lead to the activation of the relevant target genes and subsequent cell death. As mentioned above, many methods have been tried to reduce the accumulation of drugs in the cochlea and affect the ototoxicity process to mitigate the loss of hair cells. This review focuses on drug and gene therapies to reduce the ototoxicity of aminoglycosides by regulating programmed cell death, including apoptosis, autophagy, and ferroptosis. In recent decades, the increasing understanding of apoptosis, autophagy, and ferroptosis has led to the development of clinical treatments for hearing loss. The occurrence and development of ototoxicity may involve multiple forms of hair cell death and related signaling pathways, audiologists have been trying to understand how these pathways map and integrate. To better protect hair cells from ototoxic drugs, we need to understand the pathogenesis of hearing loss, multi-target, and multi-pathway therapies should be considered according to the characteristics of programmed cell death. CRISPR/Cas9 gene editing technology, which allows precise editing of target genes, has set off a new wave of research and made remarkable achievements in the treatment of drug-induced deafness. In the future, the problem of drug ototoxicity will gradually dissipate with the continuous exploration of researchers.
